# Supplementary material for: The Microtubule Destabilizer Eribulin Synergizes with STING Agonists to Promote Antitumor Efficacy in Triple-Negative Breast Cancer Models
Source: Cancers (Basel). 2022 Dec 2;14(23):5962. doi: 10.3390/cancers14235962 (PMC9740651; doi:10.3390/cancers14235962)

Figure S1

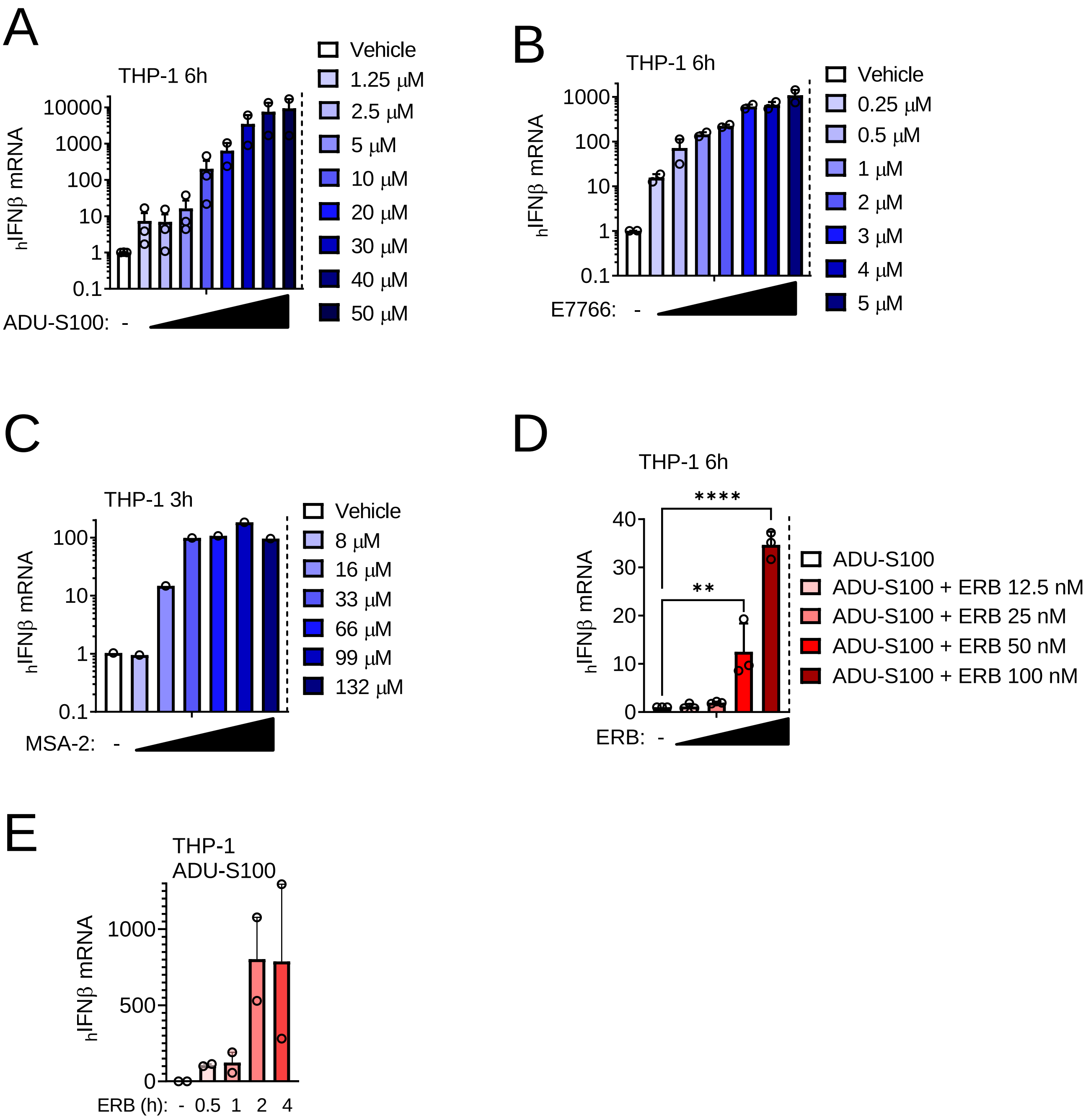

Figure S1. Concentration-dependence of STING agonists and eribulin on IFNβ expression in THP-1 cells. Human *IFNβ* mRNA expression in THP-1 cells treated with indicated concentrations of (**A**) ADU-S100 for 6 h, (**B**) E7766 for 6 h, or (**C**) MSA-2 for 3 h. (**D**) Human *IFNβ* mRNA expression in THP-1 cells treated with 10 µM ADU-S100 and indicated concentrations of eribulin (ERB) for 6 h. (**E**) Human *IFNβ* mRNA expression in THP-1 cells treated with 10 µM ADU-S100 for 6 h in combination with 100 nM ERB added for the indicated duration during the 6 h ADU-S100 treatment. Significance of eribulin determined by one-way ANOVA with Dunnett's posthoc test comparing all conditions to ADU-S100 alone. $^{**}p<0.01$, $^{****}p < 0.0001$.

Figure S2

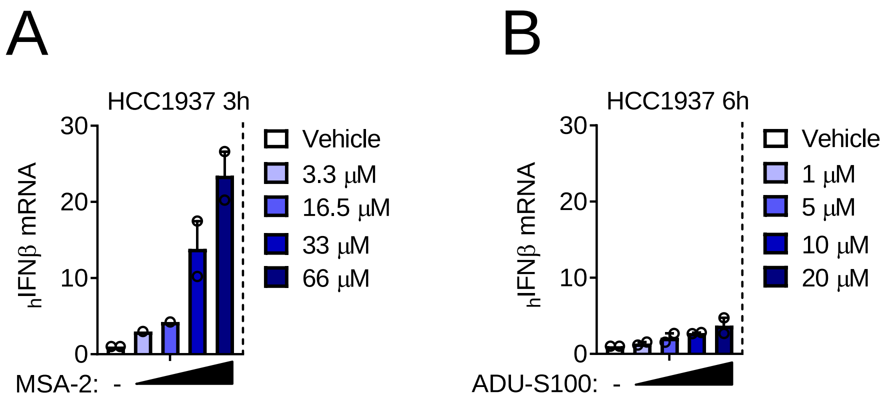

Figure S2. Concentration-dependence of STING agonists on IFNβ expression in HCC1937 TNBC cells. Human *IFNβ* mRNA expression in HCC1937 cells treated with indicated concentrations of (**A**) MSA-2 for 3 h or (**B**) ADU-S100 for 6 h.

Figure S3

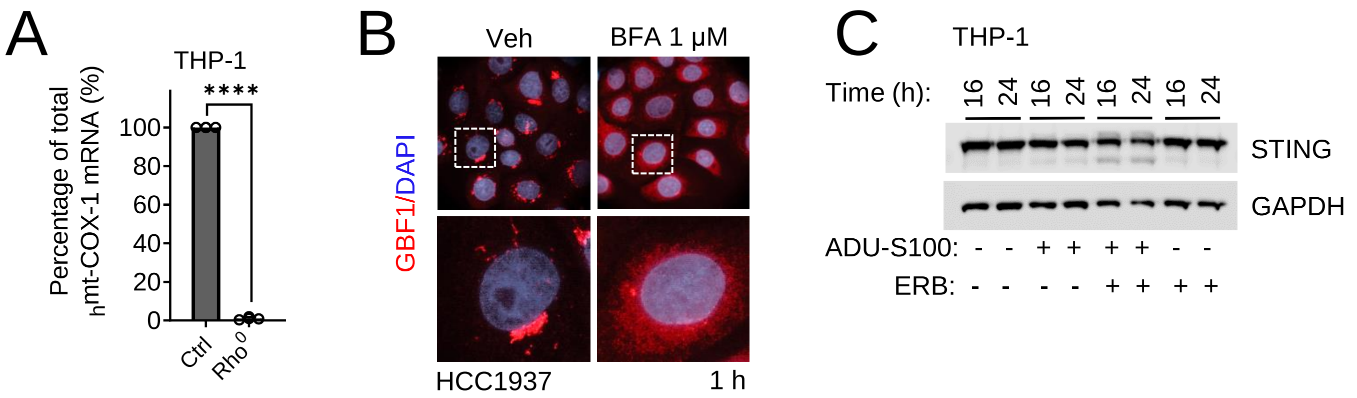

Figure S3. Eribulin's effect on the canonical STING signaling pathway. (**A**) Expression of the mitochondrially-encoded human cytochrome c oxidase subunit 1 (*COX-1*) mRNA in control and ethidium bromide (EtBr) cultured THP-1 cells (Rho^0). Significance is determined by paired t-test. ****$p < 0.0001$. (**B**) Immunofluorescence images of the cis-Golgi marker GBF1 and DAPI in HCC1937 cells treated with 1 μM BFA for 1 h. The white box insert represents an enlarged representative cell for detail, below. (**C**) Immunoblot of STING and GAPDH in THP-1 cells treated with 10 μM ADU-S100 with or without 100 nM ERB for 16 or 24 h.

Figure S4

STING
Cell Lines: THP-1, HCC1937, HCC1806, MDA-MB-453
MW: 35 kDa

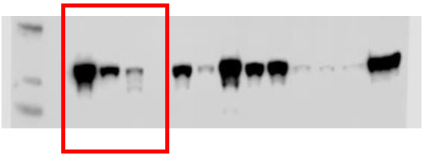

GAPDH
Cell Lines: THP-1, HCC1937, HCC1806, MDA-MB-453
MW: 36 kDa

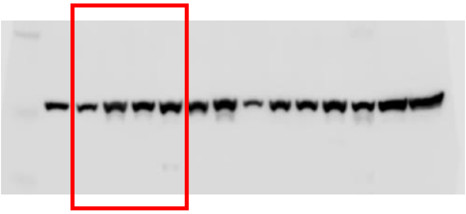

TBK1
Cell Line: THP-1
MW: 84 kDa

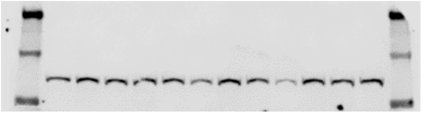

IRF3
Cell Line: THP-1
MW: 47 kDa

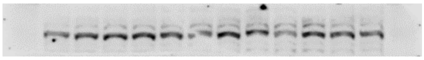

P-TBK1
Cell Line: THP-1
MW: 84 kDa

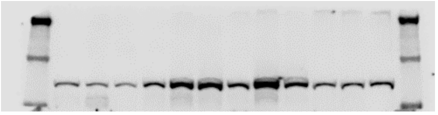

P-IRF3
Cell Line: THP-1
MW: 47 kDa

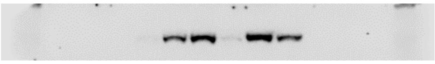

GAPDH
Cell Line: THP-1
MW: 36 kDa

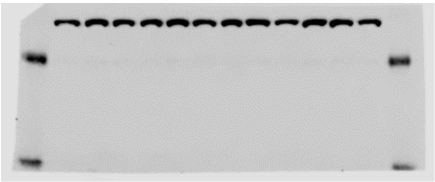

P-STING
Cell Line: THP-1
MW: 40 kDa

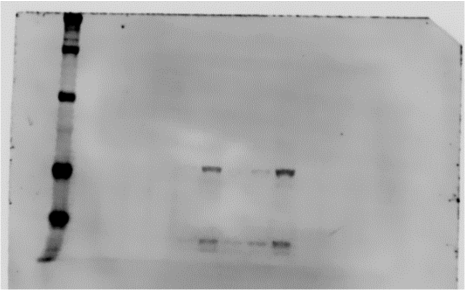

Total STING
Cell Line: THP-1
MW: 35 kDa

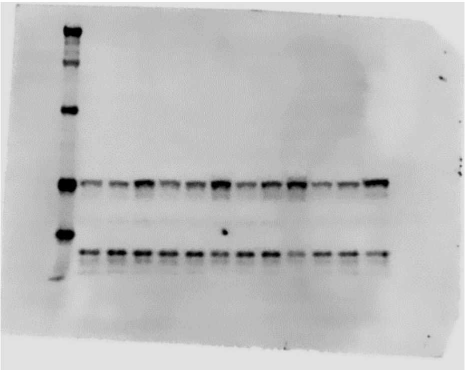

GAPDH
Cell Line: THP-1
MW: 36 kDa

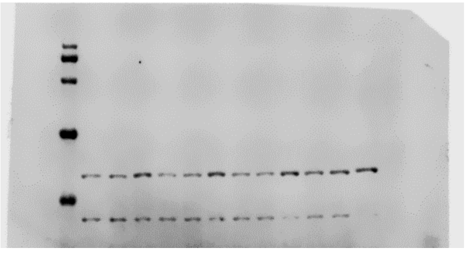

Total STING
Cell Line: HCC1937
MW: 35 kDa

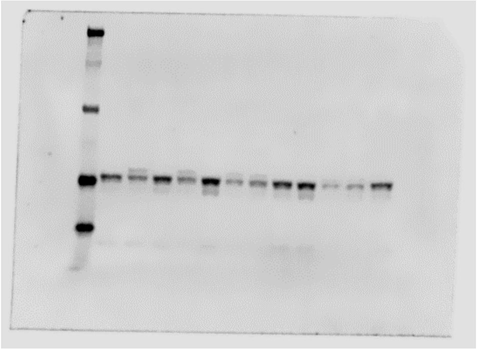

GAPDH
Cell Line: HCC1937
MW: 36 kDa

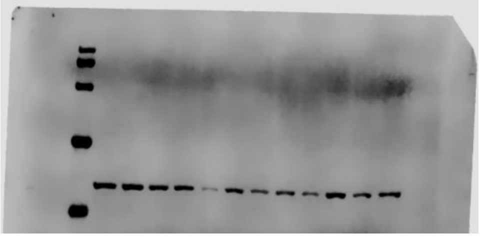

Supplement: Supplementary file 1 [file cancers-14-05962-s001.zip › cancers-2063592-supplementary.pdf]
